# Functional diversity of bacterial microbiota associated with the toxigenic benthic dinoflagellate *Prorocentrum*

**DOI:** 10.1371/journal.pone.0306108

**Published:** 2024-07-16

**Authors:** Miguel A. Martínez-Mercado, Allan D. Cembella, Edna Sánchez-Castrejón, Anaid Saavedra-Flores, Clara E. Galindo-Sánchez, Lorena M. Durán-Riveroll

**Affiliations:** 1 Departamento de Biotecnología Marina, Centro de Investigación Científica y Educación Superior de Ensenada B.C., Ensenada, Mexico; 2 Department of Ecological Chemistry, Alfred-Wegener Institut, Helmholtz-Zentrum für Polar-und Meeresforschung, Bremerhaven, Germany; 3 CONAHCyT-Departamento de Biotecnología Marina, Centro de Investigación Científica y de Educación Superior de Ensenada, B.C. Ensenada, Mexico; Stazione Zoologica Anton Dohrn, ITALY

## Abstract

Interactions between bacterial microbiota and epibenthic species of the dinoflagellate *Prorocentrum* may define the onset and persistence of benthic harmful algal blooms (bHABs). Chemical ecological interactions within the dinoflagellate phycosphere potentially involve a complex variety of organic molecules, metabolites, and toxins, including undefined bioactive compounds. In this study, the bacterial diversity and core members of the dinoflagellate-associated microbiota were defined from 11 strains of three epibenthic *Prorocentrum* species, representing three geographically disjunct locations within Mexican coastal waters. Microbiota profiles in stable monoclonal *Prorocentrum* cultures were obtained by sequencing amplicons of the V3-V4 region of the 16S rRNA gene. Thirteen classes of bacteria were identified among dinoflagellate clones, where Alphaproteobacteria, Gammaproteobacteria, and Bacteroidia were consistently dominant. The bacterial community structure exhibited significantly different grouping by the location of origin of dinoflagellate clones. No significant diversity difference was found among free-living or unattached bacteria in the dinoflagellate culture medium (M) compared with those in closer association with the dinoflagellate host cells (H). Twelve taxa were defined as core members of the bacterial assemblage, representing the genera *Algiphilus*, *Cohaesibacter*, *Labrenzia*, *Mameliella*, *Marinobacter*, *Marivita*, *Massilia*, *Muricauda*, *Roseitalea*, and an unclassified member of the Rhodobacteraceae. The core members are inferred to significantly contribute to primary and secondary metabolic functions, but no direct correlation with dinoflagellate toxigenicity was apparent. Overall the bacterial profile and implied gene functionality indicated a suite of positive interactions, suggesting either mutualism or commensalism with the dinoflagellate. The further characterization and interpretation of specific gene functions and interactions between bacteria and dinoflagellates, such as epibenthic members of genus *Prorocentrum*, are key to understanding their role in toxigenesis and bHAB development.

## Introduction

Marine species of the dinoflagellate genus *Prorocentrum* Ehrenberg are globally distributed, although epibenthic ecotypes are more prominent in subtropical and tropical regions [1]. The genus *Prorocentrum* comprises >80 defined morphospecies [2], but many morphotypes, particularly among the benthic contingent, remain only provisionally classified within various species complexes [3]. Recent efforts to resolve taxonomic and phylogenetic affinities, e.g., within the *P*. *lima* species complex, have been directed to target sequences of the 18S or 28S ribosomal RNA (rRNA) gene and internal transcribed spacer (ITS) region [4, 5].

In shallow coastal waters, *Prorocentrum* cell aggregates live attached to macroalgae, seagrasses, or other substrates, where cell proliferation can cause benthic harmful algal blooms (bHABs) [1, 4]. Recent attention on the ecological and human health consequences of bHABs has heightened scientific and biomedical awareness of these phenomena and their consequences [6]. In the benthos or attached to floating or suspended substrates, these *Prorocentrum* aggregates may negatively affect marine microfauna grazing directly upon the surfaces or living in the benthos. There is also potential for transfer of diarrhetic shellfish toxins (DSTs) produced by certain epibenthic and epiphytic *Prorocentrum* species [4] from dislodged cells to the water column [7, 8]. The relative impact of such toxigenic blooms on toxin accumulation and species interactions in benthic food webs, however, remains poorly defined.

Epibenthic dinoflagellates are naturally exposed to associated bacteria and other microeukaryotes within two broad compartments—the free-living and substrate-attached communities, respectively. Chemical interactions can occur in the extracellular space surrounding the dinoflagellate cells, denoted as the phycosphere [9], into which metabolites and other dissolved organics can diffuse and be exchanged with the aqueous medium. Chemically-mediated interactions with bacteria within the phycosphere could be beneficial or detrimental to the epibenthic dinoflagellate, or even mutually beneficial for both the dinoflagellate and the microbiota. In the mutually beneficial case, these interactions may involve the excretion or leakage of dissolved organic matter and shedding of nutrient-rich particular matter by the dinoflagellate in reciprocal exchange for vitamins or organic nutrients generated by the bacterial assemblage.

In negative interaction scenarios, “toxins” or other allelochemicals generated by the dinoflagellate may limit parasitism, colonization, or reduce competition for inorganic nutrients from the bacterial or microeukaryotic component in the microbiota [10]. Many epibenthic *Prorocentrum* species produce a suite of bioactive polyketide metabolites [11], including the DSTs responsible for diarrhetic shellfish poisoning (DSP). Production of toxic polyketides or other allelochemicals by *Prorocentrum* may be a functionally adaptative response–a chemical warfare strategy–particularly effective for epibenthic dinoflagellates as a defense against predators, and bacterial and microeukaryotic competitors for attachment space and nutrients [12]. Knowledge of the strategic importance of the bacterial community in toxin production or regulation in benthic dinoflagellates is scarce. While the toxin functions are still unknown, they may involve profound interactions with associated bacteria within the phycosphere and upon epibenthic substrates. Elucidation of the bacterial community composition and functional diversity is, therefore, the first step in defining these interactions.

In early studies of bacterial interactions with dinoflagellates, diversity was determined by culturing co-occurring bacteria [13, 14] and then by characterization of morphological or metabolic criteria and growth tolerance regimes. Later characterization studies for bacterial species in dinoflagellate cultures have been based on nucleic acid sequencing of a genetic marker, e.g. one or several hypervariable regions of the 16S rRNA gene [15, 16]. By combining morphological and sequencing approaches, it has been possible to demonstrate the influence of co-cultured bacteria associated with *P*. *lima* on growth, and toxin production rate and toxin cell quota of the dinoflagellate [16]. Such culture-based approaches allow detailed descriptions of specific bacterial members of the consortium but are limited in scope and biased for microbiome diversity studies because they are restricted to those taxa that may be readily co-cultured under defined laboratory conditions.

An alternative culture-independent approach to bacterial diversity can be obtained by high-throughput sequencing (HTS) of hypervariable regions of the 16S rRNA gene. In this way, the microbiome of several microeukaryotes (“phytoplankton”) has been characterized within a broader context, including species of planktonic dinoflagellates, such as *Margalefidinium* (= *Cochlodinium*) *polykrikoides* [17] and *Prorocentrum minimum* [18], and toxigenic benthic *Gambierdiscus balechii* [19].

The goal of this study was to evaluate the bacterial community and core membership in xenic *Prorocentrum* cultures by HTS sequencing of the V3-V4 regions of the bacterial 16S rRNA gene. The composition, diversity, and structure of the bacterial communities associated with two operationally defined fractions were compared among 11 strains of epibenthic *Prorocentrum* of differing toxigenic capacity and from distinct geographical origins from the coasts of Mexico. The comparative aspects focused primarily on *P*. *lima* because *P*. *hoffmannianum* and *P*. *rhathymum* were each represented by only a single strain. Core members of the *Prorocentrum* bacterial community were defined from fractions directly associated with the host dinoflagellate cells. The potential contribution of the core bacterial contingent to functional diversity interactions was explored by metabolic inference analysis.

## Materials and methods

### Collection and culture of *Prorocentrum* strains

Clonal cultures of epibenthic *Prorocentrum* were initiated from field material collected from coastal sites in the southern Gulf of California (Bahía de La Paz, Baja California Sur), the central Gulf of Mexico (Veracruz Reef System, Veracruz) and the Caribbean coast of Mexico (Puerto Morelos, Quintana Roo) (Table 1). Epibenthic dinoflagellates were harvested from various substrates, including seagrasses (Tracheophyta), macroalgae (Rhodophyta, Ochrophyta, and Chlorophyta), and from a rope attached to a buoy. All substrate samples (<5 g) were collected from non-protected species, and from public areas for which permits are not required for Mexico. Nevertheless, collection by investigators from the lead institute (CICESE, Ensenada) followed standard careful field practices to minimize ecosystem impacts. Microbiological research in the laboratory did not involve human or animal subjects, or cultivation of pathogenic microbes, and thus no specific endorsement from a CICESE or CONAHCYT ethics panel was necessary. Field samples were processed to establish clonal dinoflagellate strains according to previously described methods [4]. The taxonomic assignments and species identification of the *Prorocentrum* strains for the present study (Table 1) were previously determined by morphological analysis by microscopy and sequence analysis of the ITS1-5.8S_rRNA-ITS2 and 28S large-subunit (LSU) rRNA gene operon, complemented with secondary structure modeling of ITS2 rRNA variation based on compensatory base changes [4].

**Table 1:** Species, strain number, region and locality of orgin, geographical coordinates, substrate type and sampling date for collection of *Prorocentrum* and associated natural bacteria.

Species	Strain	Region of origin	Locality of origin	Geographical coordinates	Substrate type	Sampling Date
*P*. *lima*	PA02	Gulf of Mexico	Isla Verde, Veracruz Reef System, Veracruz	19°11’54.10"N, 96° 4’0.70"W	*Thalassia testudinum* (Tracheophyta)	26-May-17
*P*. *lima*	PA08					26-May-17
*P*. *lima*	PA09				*Laurencia* sp. (Rhodophyta)	21-Jul-17
*P*. *lima*	PA61		Piedra de la Virgen, Veracruz Reef System, Veracruz	19°13’5.93"N, 96° 2’52.19"W	*Padina* sp. (Ochrophyta)	13-May-18
*P*. *lima*	PA78				Brown algae species mix (Ochrophyta)	13-May-18
*P*. *hoffmannianum*	PA85					13-May-18
*P*. *rhathymum*	PA20	Mexican Caribbean	Puerto Morelos, Quintana Roo	20°50’48.55"N, 86°52’30.53"W	Buoy-attached Rope	29-Oct-17
*P*. *lima*	PA26				*Sargassum* sp. (Ochrophyta)	29-Oct-17
*P*. *lima*	PA44	Gulf of California	Bahía de La Paz, Baja California Sur	24°09’30.01’’N, 110°19’12.10’’W	Sediment w/dead brown macroalgae	03-Jan-18
*P*. *lima*	PA49				*Ulva* sp. (Chlorophyta)	03-Jan-18
*P*. *lima*	PA53				*Sargassum* sp. (Ochrophyta)	03-Jan-18

In brief, for the current experiments, xenic monoclonal *Prorocentrum* cultures were grown in 60x15 mm plastic Petri plates on 50%-strength GSe growth medium [20], modified without soil extract supplement. Dinoflagellate cultures were incubated at 25 ± 1 °C on a 12:12 h light:dark cycle and under illumination of 50 μmol photons m^-2^ s^-1^. Exponentially growing *Prorocentrum* were transferred into 125 mL Erlenmeyer flasks for further growth on full-strength GSe medium under the same environmental conditions and harvested after 3 to 4 weeks.

### Harvest and fractionation of dinoflagellate-associated bacteria

Dinoflagellate-associated bacteria were harvested from the *Prorocentrum* cells and growth medium by gentle sieving, with repeated washing with sterile seawater, through a sterile 20 μm-nylon mesh. Dinoflagellate cells with surface-attached and intracellular (e.g., endosymbiotic) bacteria, hereafter comprising the dinoflagellate host (H) microbiome (S1 Fig), were recovered from the upper mesh surface by gentle backwashing with sterile-filtered seawater into 15 mL centrifuge tubes. After centrifugation (524R Eppendorf, Hamburg, Germany) at 6,000 × *g* at 4 °C for 3 min, the supernatant was discarded, and the H-fraction pellets were transferred with sterile seawater into 2 mL microtubes, and re-centrifuged as above. Bacteria from the filtrate passing through the 20 μm-nylon mesh, containing free-living or loosely bound dinoflagellate-associated bacteria, were harvested as the medium (M)-microbiome (S1 Fig) by centrifugation (Megafuge 40R, ThermoScientific, Waltham, USA) (15,000 × *g* at 4 °C for 15 min) of 100 mL filtrate. After discarding the supernatants, the bacterial cell pellets of M-fractions were transferred into 2 mL microtubes. The pellets of H- and M-fractions were frozen at -20 °C until DNA extraction.

### Amplification and sequencing of bacterial 16S rRNA gene

Total DNA was extracted with the DNeasy Power Soil Kit (QIAGEN, Hilden, Germany) following the manufacturer’s instructions, with quantification by NanoDrop 2000 spectrophotometer (Thermo Scientific, Waltham, MA, United States). Due to high biomass contribution of dinoflagellate cells to the H-fraction, approximately 100 mg of H-samples versus 10 mg of M-fractions were extracted for amplification of the 16S rRNA bacterial genes. Samples were homogenized in extraction solution provided in the DNA extraction kit (QIAGEN, Hilden, Germany) in a FastPrep-24^®^ 5G instrument (MP Biomedicals, Santa Ana, CA, United States) over three cycles of 30 s at 6.0 m s^-1^; each cycle was followed by a 5 min incubation on ice to minimize thermal degradation.

DNA library preparation was based on the MiSeq 16S Metagenomic Sequencing Library Preparation B protocol (Illumina, San Diego, CA, United States), with modifications according to the respective primers. The hypervariable regions V3-V4 of the 16S rRNA gene were amplified using the primer sequences 341F/785R published by Klindworth et al. [21]. PCR reactions were performed in duplicate in a final volume of 20 μL reaction mix: 1X KAPA HiFi Buffer GC, 2.5 mM MgCl_2_, 0.3 mM KAPA dNTP Mix, 0.3 μM of each primer, 1 U of KAPA HiFi HotStart DNA Polymerase (KAPA Biosystems, Wilmington, United States) and 12.5 ng of total DNA. The thermal cycler program was: 3 min at 95 °C, 25 cycles of 30 s at 95 °C, 30 s at 55 °C, and 30 s at 72 °C, with a final extension of 5 min at 72 °C.

PCR products were visualized in 2% agarose gel and then purified with Agencourt AMPure XP paramagnetic beads (Beckman Coulter, Brea, CA, United States). The sequencing Illumina indexes were incorporated for dual indexing with the Nextera XT index kit according to the manufacturer’s instructions. The libraries were quantified with a Qubit^™^ dsDNA High Sensitivity assay in a Qubit^®^ 3.0 fluorometer (Thermo Scientific, Waltham, MA, United States). Each library was normalized at 40 nM, and then pooled and diluted to 4 nM. Paired-end reads of 250 bp were generated on a MiSeq sequencing platform (Illumina, San Diego, CA, USA).

### Sequencing data analysis

Sequencing reads were processed with the dada2 v.1.14 package [22] in R v3.6 [23] following the author’s recommendations and with the necessary parameter adjustments. Sequence processing began by removing primer sequences from both reads, followed by base quality filtering, error rate calculation, and dereplication of reads. Quality-controlled reads were then denoised with the dada2 algorithm. The consensus method was used to identify and remove chimeras. Afterward, successfully merged paired reads were interpreted to define Amplicon Sequence Variants (ASVs). ASV sequences were taxonomically assigned with the ‘assignTaxonomy’ function at a confidence threshold of 80 and the SILVA 138.1 database [24] formatted for dada2 (https://doi.org/10.5281/zenodo.4587955). Packages DECIPHER v.2.14.0 [25], and phangorn v.2.5.5 [26] were used to align ASV sequences based on an initial single-linkage guide tree constructed from distances on shared k-mers.

A neighbor-joining (NJ) tree was constructed as the starting point for Maximum Likelihood (ML) tree inference based on a GTR+I+G model. ASV count table, taxonomy assignment, and ML tree were combined in a phyloseq object (v.1.30.0) [27] for phylogenetic and ecological analysis in R. Sequence counts were randomly subsampled to the same depth (91,115 reads), corresponding to the minimum deep-sequencing among samples. UniFrac distances (weighted and unweighted) among samples were used to explore the bacterial community structure by Principal Coordinates Analysis. Evaluation of sample grouping by location of origin was tested by PERMANOVA (9,999 permutations; α = 0.05) and validated by analysis of the variance of the groups’ dispersions. Several alternative alpha diversity indexes—observed ASVs (richness), Shannon diversity index, and Faith’s phylogenetic distance—were compared between dinoflagellate host (H)- and medium (M)- bacterial communities by a Kolmogorov-Smirnov test (α = 0.05). Community composition was explored at the class level by summarizing sequence counts per sample and plotting taxa with relative abundance >0.1%. Shared genera between the H and M bacteria were visualized in a Venn diagram. Core members were defined as those ASVs present in >75% of the samples with a minimum relative abundance of 0.0001% per sample, as for the endosymbiotic dinoflagellate *Symbiodinium* [28].

Prediction of the presence and abundance of the Kyoto Encyclopedia of Genes and Genomes (KEGG) metabolic pathways [29] from ASVs of the host microbiome was conducted with PICRUSt2 v.2.2.0_b stratified pipeline, using a Nearest Sequenced Taxon Index (NSTI) < 0.2 [30], by the phylogenetic placement of short sequences to identify ancestral states [31–33]. The inferred metabolic profiles are used to support and complement known functions of the core members by comparing their contribution versus the contribution of non-core members through a Mann-Whitney test (α = 0.05) with Bonferroni correction for multiple testing.

### Analysis of dinoflagellate DST composition in relation with associated bacteria

The toxin composition and cell quota of DSTs of toxigenic strains of *P*. *lima* and one of *P*. *hoffmannianum* were determined from H-fraction samples extracted in 50% MeOH and analyzed by liquid chromatography coupled with tandem mass spectrometry (LC-MS/MS) by previously published methods [4, 16]. Identification of bacterial taxa whose abundance correlated with the toxin production was tested by Pearson correlation and corrected for multiple testing by False Discovery Rate (FDR< 0.05) with the microbiome package [34]. The possible association of metabolic functions related to DST production was restricted to the 10 toxigenic strains of *P*. *lima* and *P*. *hoffmannianum*, leaving out the non-toxigenic *P*. *rhathymum* (PA20) [4], represented by only a single strain.

## Results

The microbiome associated with cultured strains of the dinoflagellate genus *Prorocentrum* among three species (*P*. *lima*, *P*. *rhathymum*, and *P*. *hoffmannianum*) from disjunct geographical regions and collected from different substrates revealed high molecular diversity in both the dinoflagellate host (H)- and medium (M)-fractions. High-throughput sequencing of the bacterial community targeted to the V3-V4 regions of the 16S rRNA gene generated an average of 15.4 x 10^4^ reads for H-fractions and 14.1 x 10^4^ reads for M-fractions. After quality control and taxonomic filtration, reads analysis yielded an average of 58 bacterial ASVs in H-fractions and 67 in M-fractions (S1 Table). The sequencing depth achieved was sufficient to obtain a good representation of the diversity of each sample, as observed by rarefaction curves approaching the asymptote (S2 Fig).

### Composition of the bacterial community associated with *Prorocentrum*

**Fig 1:**
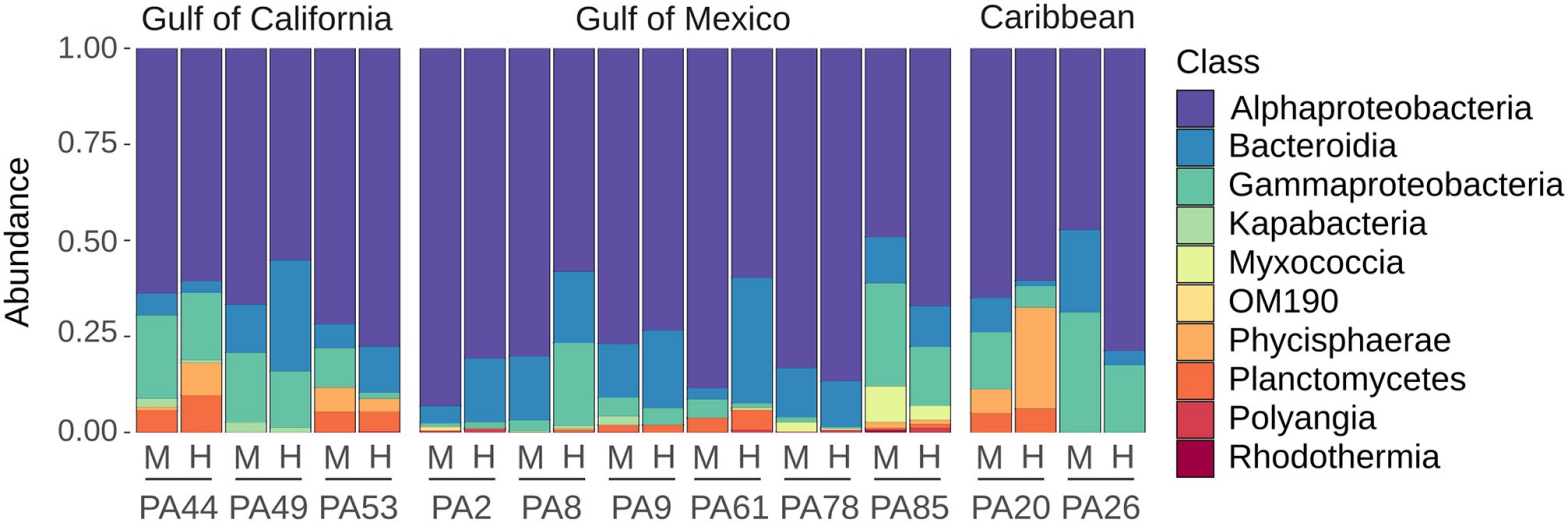
Composition of the bacterial community in *Prorocentrum* cultures. Bacterial profiles were analyzed by sequencing of V3-V4 16S rRNA gene amplicons. Relative abundances per sample are shown at the class level for abundant bacterial taxa (>0.1%) in the fraction associated with the host (H) or free-living in the culture medium (M) of nine strains of *P*. *lima* and one strain each of *P*. *rhathymum* (PA20) and *P*. *hoffmannianum* (PA85). Location of dinoflagellate strain origin is indicated above in the figure and in Table 1.

A total of 14 classes from eight phyla were represented among the ASVs from the H- and M-fractions. At the class level, 10 were considered dominant as they were represented in relative ASV abundances higher than 0.1%. Alphaproteobacteria was the most abundant class (relative abundances of 47 to 93%) in both fractions (Fig 1); there was no significant difference in the dominance structure except for a switch in ranking order from the third to the fourth position of Gammaproteobacteria and Phycisphaerae between H- and M-fractions, respectively. The classes Bacteroidia and Gammaproteobacteria were present in all H- and M-fractions, but classes Phycisphaerae and Planctomycetes were relatively abundant (>5%) in only seven and eight samples, respectively (Fig 1). The least abundant ASV classes were represented by Actinobacteria, Bacilli, Desulforomonadia, and KD4-96.

An exploration of the taxonomic distribution at the genus level (S3 Fig) showed that the dominant classes were each represented by several genera, e.g. *Labrenzia*, *Roseitalea*, *Cohaesibacter*, *Marivita*, *Thalassobaculum* (among others) for Alphaproteobacteria; and *Marinobacter*, *Massilia*, *Algiphilus*, and generically unassigned-Salinisphaerales for Gammaproteobacteria. Bacteroidia were represented by only two genera: *Muricauda* and *Phaeodactylibacter*, with the former yielding more ASVs from both fractions among the dinoflagellate cultures. Seventy-three percent of genera (69 out of 95) were shared between the two bacterial fractions; only 20 genera (21%) were exclusively found in M-fractions and six genera (6%) only in H-fractions. The only non-toxigenic strain included in this study, *P*. *rhathymum* PA20, exhibited distinctive bacterial genera in higher abundance compared to all toxigenic *Prorocentrum* strains. In decreasing order of abundance, these genera were: SM1A02 (Phycisphaerales), *Hoeflea* (Alphaproteobacteria), *Methylophaga* (Gammaproteobacteria), and *Roseicyclus* (Alphaproteobacteria).

### Core members of the bacterial community

The core members of the dinoflagellate-associated bacterial community were defined (as ASVs) by focusing only on the H-fractions. These H-bacteria are physically closer to the dinoflagellate cells, thereby establishing continuous and more intimate interactions than with free-living bacteria in the medium (M). Furthermore, there was no evidence from comparing H- and M-fractions of significant differences in compositional structure at the class level. Core membership defined by taking into account only the toxigenic *Prorocentrum* strains yielded 12 bacterial ASVs that met the criteria as core members (S4 Fig). Notably, eight core-ASVs were found in all samples.

**Fig 2:**
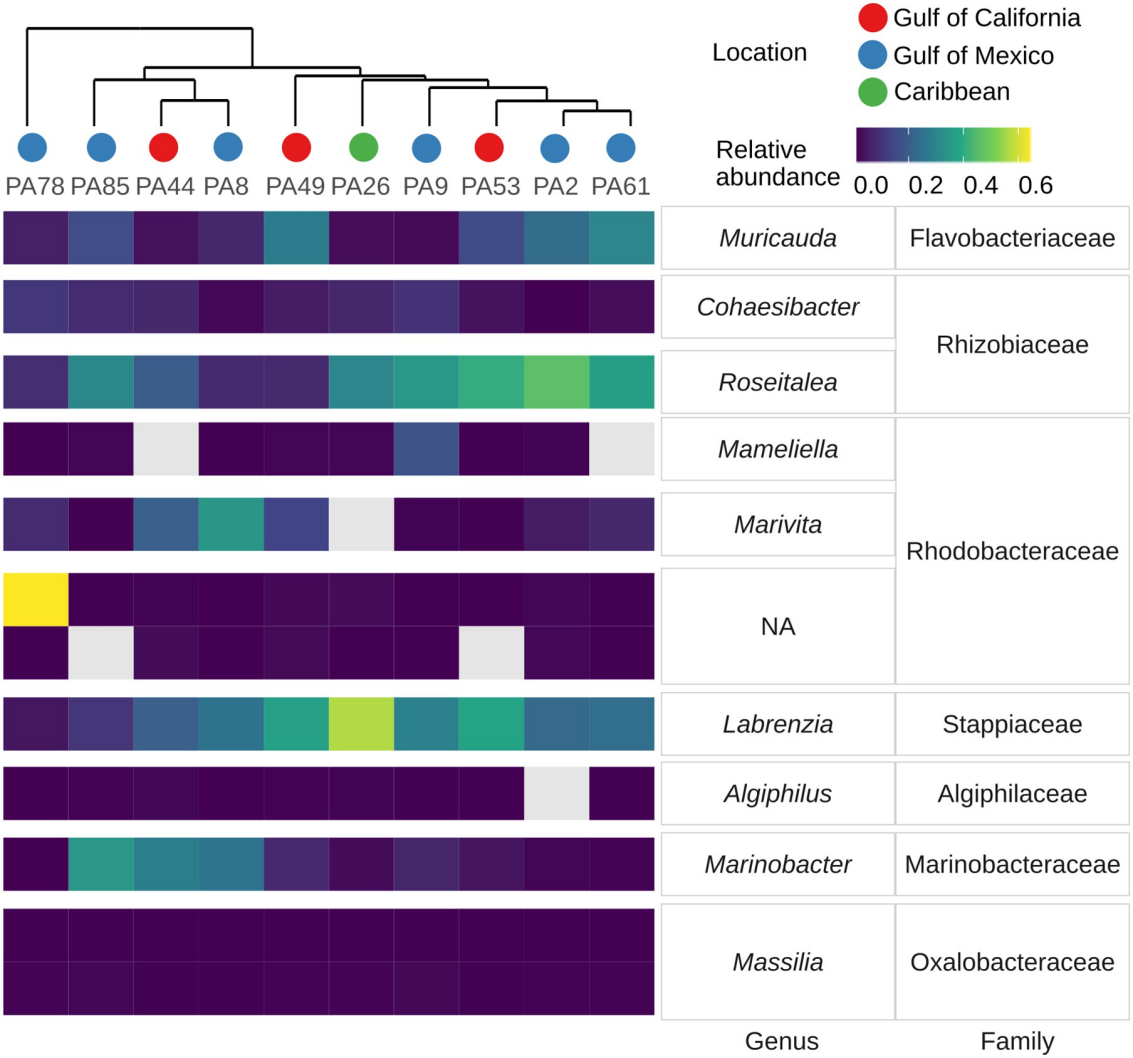
Relative ASV abundance of core bacterial members co-occurring with *Prorocentrum* strains. Heatmap shows core ASVs and their associated taxonomy (at family and genus levels) per sample. Dendrogram of hierarchical cluster analysis of samples is shown at the top.

The defined bacterial core members associated with *Prorocentrum* belonged to three classes (Alphaproteobacteria, Gammaproteobacteria, and Bacteroidia), seven families, and 10 genera (Fig 2). The core member assigned to genus *Labrenzia* had a consistently high abundance, except for *Prorocentrum* strain PA78. Other genera represented by ASVs with high abundance across samples were *Roseitalea*, *Muricauda*, and *Marinobacter*. There were also ASVs with low abundance but high occurrence (i.e., rare taxa), representing the genera *Massilia* (2 ASVs) and *Cohaesibacter* (1 ASV). The core-ASVs belonging to *Algiphilus*, *Marivita*, *Mameliella*, and unclassified-Rhodobacteraceae were absent from a few strains: ASVs associated with *Algiphilus* were not found in *Prorocentrum* PA2, *Marivita* was absent from PA26, whilst *Mameliella* ASVs were not detected from PA44 and PA61, nor Rhodobacteraceae from PA53 and PA85. The core member ASV_004 assigned as an unclassified-Rhodobacteraceae was the most abundant taxon in strain PA78, yielding a very distinctive pattern among core members. A cluster analysis, however, indicated no apparent grouping by location of origin based only on the abundance of core members (Fig 2).

Potential metabolic functions were predicted with the ASV sequences of the H-fractions to explore the functional contribution of the core members. A reliable functional profile was inferred for all ten H-fractions, according to the NSTI-sample values in the range of 0.025 to 0.125 (S1 Table). In these profiles, the core members contribute significantly more to nine major metabolic pathways compared to the contribution of non-core members (S2 Table). The key functional subcategories significantly supported by the core members include basal metabolism of different primary carbon sources (e.g., carbohydrates, amino acids and associated cofactors and vitamins), and secondary metabolism (including biosynthesis of terpenoids and polyketides, and degradation of xenobiotics).

### Differences in bacterial community diversity among *Prorocentrum* strains

**Fig 3:**
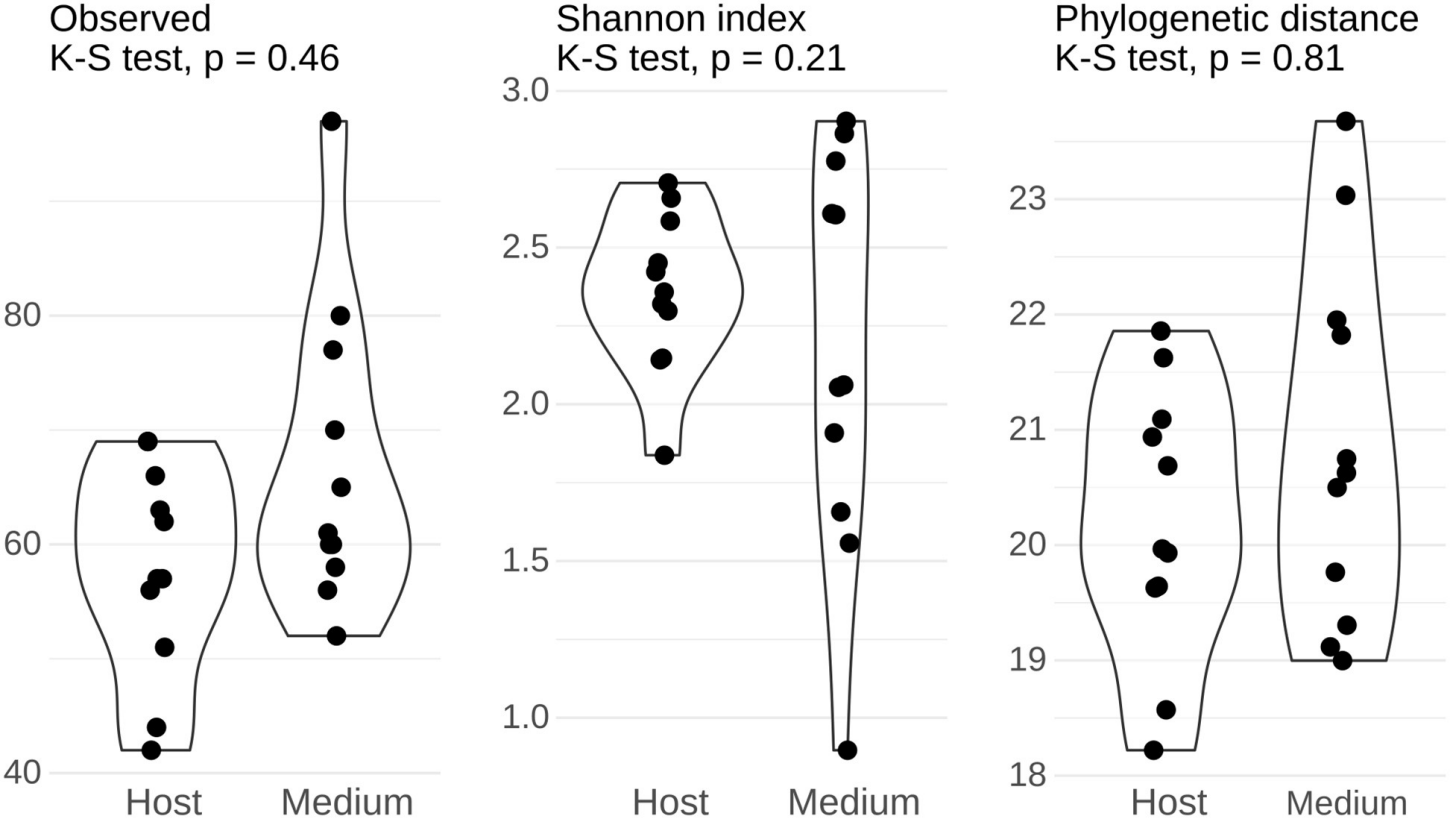
Comparison of bacterial community diversity between *Prorocentrum* culture compartments. Violin plots show the distribution of alpha diversity indices (A) observed ASVs, (B) Shannon index and (C) phylogenetic distance calculated for the bacterial community directly associated with *Prorocentrum* (Host = H) and free-living in culture medium (Medium = M). Comparison between compartments was by a Kolmogorov-Smirnov test (*α* = 0.05).

**Fig 4:**
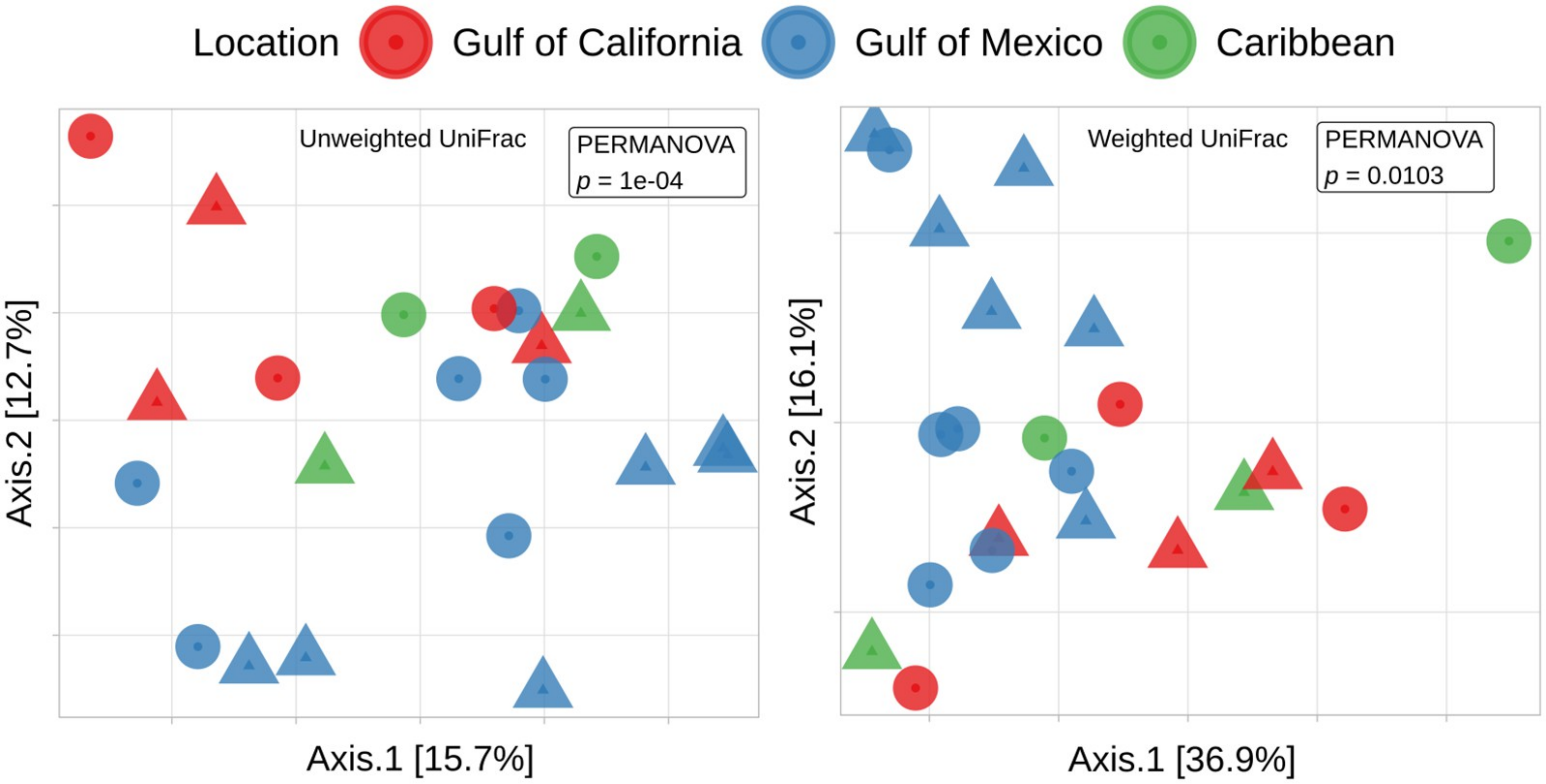
Bacterial community structure in *Prorocentrum* cultures. Principal coordinates analysis (PCA) of (A) unweighted and (B) weighted UniFrac distances among bacterial communities from *Prorocentrum* strains. Differences in community associated with the location of origin (shown by colors) were found by PERMANOVA test (α = 0.05, permutations = 9,999). Shapes indicate compartments associated directly with the host (H, circles) or free-living in the culture medium (M, triangles).

The alpha-diversity and community structure of the bacterial community in the H- and M- fractions overlap. The values of alpha-diversity indices (Observed ASVs, Shannon index, and Faith’s phylogenetic distance) compared by the Kolmogorov-Smirnov test (α = 0.05) were not significantly different between the H- and M-fractions (Fig 3). The UniFrac distance indicates that the samples group in compartments by the presence of taxa (PERMANOVA unweighted, *p* = 0.024), but not depending on the relative abundance of these taxa (PERMANOVA: weighted, *p* = 0.49, Fig 4). Nevertheless, despite the fact that the *Prorocentrum* strains were established and maintained under identical conditions, the bacterial community associated with these dinoflagellate strains was significantly different among the locations of origin: Gulf of California, Gulf of Mexico, and the Caribbean (PERMANOVA: unweighted, *p* = 1e-04; weighted, *p* = 0.0103; Fig 4).

### Effects of associated bacteria on toxigenicity of *Prorocentrum* strains

The linear polyketide okadaic acid (OA) and its respective diol-ester (OA-D8) tended to be the dominant DST analogs among all 10 toxigenic *Prorocentrum* strains considered for the bacterial community analysis herein. These two analogs typically comprised >50% of the total DST composition on a molar basis; other minor derivatives were present but varied widely among strains (S5 Fig and S1 Table). These minor variants included dinophysistoxin 1 (DTX1), its respective diol-ester (DTX1-D8), and two structurally undefined analogs, DTX1a and DTX1a-D8, in minor proportions. Strain *P*. *lima* PA53 from the Gulf of California produced mostly OA (about 75%), whereas PA49 cells from the same location yielded a similar proportion of OA and OA-D8 (<50%). In contrast, strain PA78 from the Gulf of Mexico produced mainly DTX1 (50%) but among all strains was the only one expressing such a relatively high DTX1 content.

**Fig 5:**
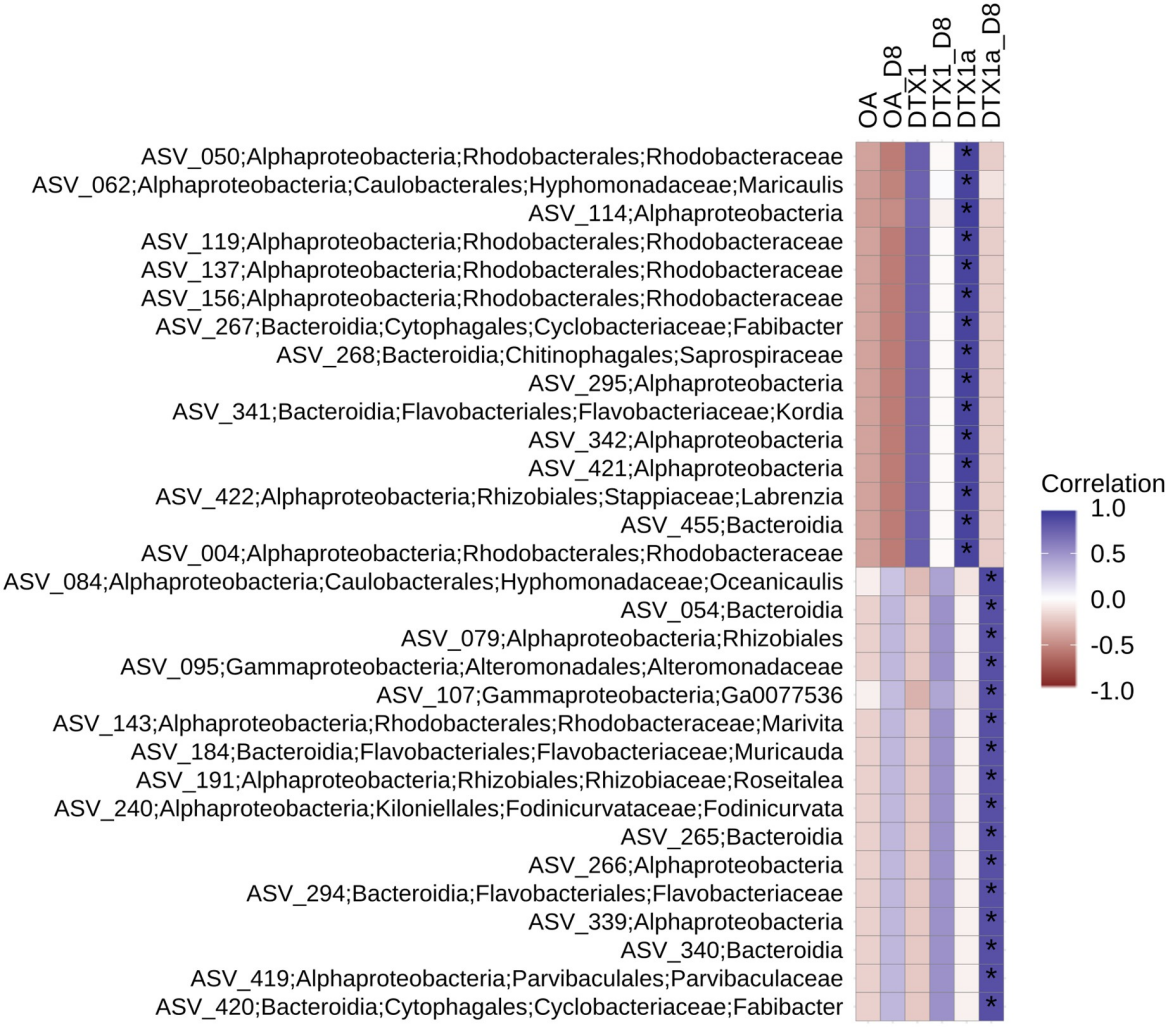
Correlation between diarrhetic shellfish toxin (DST) production of *Prorocentrum* strains and bacterial community members. Pearson correlation test with false discovery rate correction (FDR< 0.05) was calculated between the relative ASV abundance of H-associated bacteria and the relative compositional abundance (fmol cell^-1^) of DSTs produced by ten *Prorocentrum* strains. For clarity, the heatmap shows the correlations only for significant ASVs (*), but for all six DSTs determined among the strains: OA, okadaic acid; OA-D8, okadaic acid diol-ester; DTX1, dinophysistoxin 1; DTX1-D8, dinophysistoxin 1 diol-ester; DTX1a, undescribed DTX1 isomer; DTX1a-D8, undescribed DTX1a isomer.

The relationship between the relative abundance patterns of the bacterial community and DST production of dinoflagellate strains was evaluated by Pearson correlation analysis to identify bacterial taxa associated with the toxigenicity of studied strains. The members of the H-fractions showed a significant correlation with two of six DST analogs produced by the analyzed *Prorocentrum* strains (Fig 5): dinophysistoxins DTX1a and DTX1a-D8 showed a significant positive correlation (Pearson correlation *r* with FDR correction <0.05) with a set of 15 and 16 ASVs, respectively. Among these, only ASV_004 (an unclassified Rhodobacteraceae) belongs to the core members. Considering the taxa assigned to correlate ASVs with DST composition, four other genera were also significantly related to the core group: *Labrenzia* with DTX1a; and *Marivita*, *Muricauda*, and *Roseitalea* with DTX1a-D8. Of note, no significant correlations were found between members of the bacterial community and the other DSTs produced by *Prorocentrum* (OA, OA-D8, DTX1, DTX1-D8), and negative correlations were not significant.

## Discussion

### Composition of the bacterial community

Alphaproteobacteria dominated the bacterial community of the *Prorocentrum* strains, and was the dominant class among all samples of both H- and M-compartments analyzed. Alphaproteobacteria, together with Bacteroidia and Gammaproteobacteria are also the dominant lineages of microbial communities in natural planktonic phytoplankton blooms [35], and are the most common classes represented in the microbiome of benthic dinoflagellates [16, 19, 36]. Other classes with abundance >0.1% identified as part of *Prorocentrum* microbial community are worthy of future research because most of them (Phycisphaerae, Planctomycetes, Polyangia, Myxococcia, and Rhodothermia) occur in similar or even higher proportions in the microbiota of other epibenthic dinoflagellates, such as *Gambierdiscus australes*, *G*. *balechii*, *G*. *caribeaus*, *G*. *carolinianus*, or *Ostreopsis ovata* [19, 37–39]. These bacterial classes are frequently found as microbial community components of a wide variety of cultured Symbiodiniaceae–most often parasitic or endosymbiotic dinoflagellates known colloquially as members of the zooxanthellae (e.g., of corals) [40]. To our knowledge, the two exceptions to this apparent cosmopolitan distribution would be the class OM190 (phylum Planctomycetota), found only associated with cultures of various species of the dinoflagellate *Alexandrium* [41] and phaeophytes (“kelp”, e.g., *Laminaria hyperborea*) [42], and Kapabacteria (phylum Ca. Kapabacteria). The latter taxon (previously OPB56) is an extremophile whose mixed-cultured member was obtained from a bioreactor processing mining wastewater [43] and is not typically found in shallow-water marine benthic ecosystems.

*Roseobacter* was previously found as the dominant lineage at the genus level associated with benthic *P*. *lima* by bacterial plate isolation techniques [13, 14], and by Denaturing Gradient Gel Electrophoresis (DGGE) with the planktonic species *P*. *minimum* [18]. In the current HTS-bacterial community analysis, the taxa closest to *Roseobacter* were unclassified members of the parental Rhodobacteraceae family, also in agreement with a previous study by our group [16] based on a different set of *Prorocentrum* strains (except for PA2 and PA26) from the Gulf of Mexico and the Caribbean. The bacterial taxa recovered through the culturing approach in the latter study were not always among the most abundant as presented herein from HTS. For example, the highly abundant core member of the genus *Labrenzia*, commonly associated with dinoflagellates [19, 28, 38] and other eukaryotic microalgae such as diatoms [44], was not recovered in the aforementioned *P*. *lima* study. On the other hand, the genus *Marinobacter* was indeed identified by both approaches, and even as a core member in the present HTS study. In fact, the four classes identified in the previous study [16] (Actinobacteria, Flavobacteriia [herein reported as order Flavobacteriales], Alphaproteobacteria, and Gammaproteobacteria) were effectively recovered here, but along with 10 other classes, reflecting the broader scope of HTS.

### Composition and diversity of bacterial communities in compartments

In xenic cultures of benthic dinoflagellates, bacteria can inhabit two broad compartments: free-living or aggregated in the culture medium (M) or directly associated with the dinoflagellate host (H). The bacteria in H-fractions may be attached to the dinoflagellate cell surface, occur as endosymbionts, or remain bound to mucus within the phycosphere. These compartments promote different lifestyle strategies, and thus, in a natural setting, they are typically inhabited by different bacteria [45]. A deeper analysis of this relationship focused, therefore, on the host associated bacterial community (H) of toxigenic strains, as the interactions are expected to be continuous and direct, with an expected significant impact on the ecophysiology of the dinoflagellate host.

The bacterial community of the *Prorocentrum* strains was dominated by Alphaproteobacteria, and to a lesser extent, Bacteroidia and Gammaproteobacteria, in both H- and M-fractions. Among the set of 11 *Prorocentrum* strains, the bacterial diversity and composition overlapped among clones and was only differentiated by a few genera each in both the H- and M-fractions. Major differences in the bacterial community between these fractions were found in HTS-based studies of *P*. *minimum* [18] and a metagenomic analysis on dynamics of free-living and attached bacterial assemblages in the centric diatom *Skeletonema* sp. [46]. In both cases, however, the microbiomes were associated with planktonic species and may be expected to differ in structure and composition of their respective compartments because of less spatially restricted microenvironments. Culture-based studies of the microbiome associated with strains of benthic *Prorocentrum* species [14, 16, 47], however, have shown dramatic differences in composition and structure of these compartments. The inherent selectivity of culture-based methods, i.e. the unculturable microbial majority [48], may be the cause for the apparent discrepancies between HTS- and culture-based approaches to define benthic dinoflagellate microbiomes.

The alpha- and beta- diversity overlap between bacteria in the culture medium (M-fraction) and host associated (H-fraction) may arise from an expanded but more loosely defined phycosphere created under stable culture conditions. The relatively limited space in an enclosed culture vessel can result in a more homogeneous distribution of bioactive compounds and nutrients than otherwise would be available by turbulence and diffusion from the water column or from benthic substrates in the natural environment. During different growth stages, specific types of organic molecules are released by dinoflagellate cells through leakage and excretion [18, 49], and, presumably, these metabolites reach equilibrium within the phycosphere in dinoflagellate cultures. Cultured benthic dinoflagellates also frequently produce abundant polysaccharide (mucus) surrounding the cell, serving as an organic substrate for bacterial growth. In support of this interpretation, the bacteria in the M-fractions of *P*. *minimum* were reported to be considerably more affected by changes in the growth stage of the host than those in the H-fractions [18]. In such cases, bacteria do not require direct interaction with the dinoflagellate to obtain certain nutrients or receive allelochemical signals, but retain the option to interact directly with the dinoflagellate for very specific needs within defined timeframes.

### Functional dependence of locations of origin and microenvironmental selection

Factors such as specific location of origin and time of collection related to seasonal or other environmental cycles are considered to play an important role in the composition and structure of the microbiome developed in eukaryotic microalgal culture [50]. Differences in bacterial community composition related to the location of origin were also observed in the current study, but reflected only as crude biogeographical profile. Communities of marine bacteria vary latitudinally [51] and seasonally [52], yet the microbiome associated to microalgae has been determined to be conservative and species-specific [53, 54]. What may be true for phytoplanktonic assemblages that are not spatially restricted may not be valid for microbiomes associated with benthic dinoflagellates, which reflect a more stable microenvironment. For example, two benthic/epiphytic dinoflagellate species (*Gambierdiscus australes* and *Ostreopsis* cf. *ovata*) collected and isolated from same beach presented different bacterial profiles [38], but several strains of the planktonic dinoflagellate *Alexandrium ostenfeldii* collected from diverse locations in the Baltic Sea developed a similar bacterial profile in culture [41]. It is not surprising, therefore, that benthic *Prorocentrum* strains of two disjunct locations, i.e. North East Pacific Ocean and the Gulf of Mexico, exhibit a generally different bacterial community structure, but with enough common taxa to define the core members.

The bacterial compositional profile is typically reduced in diversity by selection and adaptation processes during co-culture with dinoflagellates. The “genetics of survivors” issue must also be considered from the temporal perspective–as a function of time in culture. Nevertheless, long-term cultures (since 1994) of *Ostreococcus tauri* (Chlorophyta) have exhibited the same dominant bacterial taxa (*Marinobacter*) through the different culture stages of the alga, as well as when the strains was grown under different conditions in alternative media [55]. While we are aware of the time-series selection issue in dinoflagellate culture, a relatively stable bacterial community will arise under long term growth in homogeneous conditions. Previous analyzes of the microbiome throughout the culture adaptation process in diatoms and other dinoflagellates have found that once the microalgal culture is established, the companion microbiota is stable as well [44, 50, 54, 56]. In fact, an increase in microbiome evenness was observed in the culture of diatoms over propagation time due to the reduction of the dominant taxa [44].

In the current experiments with *Prorocentrum*, cultures were maintained under uniform standardized environmental conditions designed to optimize dinoflagellate growth success, but on growth media richer in both macronutrients and micronutrients [20] than those typically found in their natural environment. Therefore, any bacteria carried over from the origin pass through the same selective factors of culture acclimation through successive transfer cycles. The bacterial profile is then an indication of the epibenthic bacterial community present in the locations of origin and also capable of surviving interaction with the dinoflagellates. The transitional selection process that inevitably occurs upon dinoflagellate isolation argues strongly for early determination of bacterial diversity by high-selectivity techniques such as HTS to minimize culture artifacts. The high cryptic bacterial diversity among multiple *P*. *lima* strains cannot be interpreted further to compare variation at the dinoflagellate species level–*P*. *hoffmannianum* and *P*. *rhathymum* are represented only by a single strain each in the current study.

### Known and potential functions of core members

The inferred metagenome serves only as a guide to focus the exploration of interactions, and thus we employ only the metabolic functional categories defined from the literature cited below. The metabolic strategies reported in similar or representative taxa related to the twelve bacterial ASVs identified as core members and their inferred metabolic contributions to interactions with the dinoflagellate may be grouped into three broad categories: stress tolerance, nutrient acquisition, and molecular bioactive interactions. The two most abundant core members *Labrenzia* and *Roseitalea* are known for the production of antioxidants. *Labrenzia* is capable of biosynthesis and degradation of dimethylsulfoniopropionate (DMS) [57]–a potent allelochemical, which may be produced as a stress response in coral holobionts. *Roseitalea* is characterized by the production of carotenoids [58]. *Muricauda* representatives produce the carotenoid zeaxanthin, suggested to protect corals from environmental stress [59], such as the high solar irradiance impacting benthic communities in shallow coastal waters. Other studies have reported the association of *Labrenzia*, *Roseitalea* and *Muricauda*, as well as *Algiphilus*, to thermo-tolerance in dinoflagellates of the endosymbiont family Symbiodiniaceae [60, 61]. The stress tolerance capabilities of these core members may be a feature leveraged by the dinoflagellate in specific environmental circumstances, thereby allowing the species to thrive in challenging environments.

The metabolic and signalling interactions between associated bacteria and eukaryotic phytoplankton have been investigated in several studies [62, 63]. Nutrient acquisition is among the central interactions. The exchange of fixed nitrogen for amino acids and organic carbon is known in terrestrial and aquatic members of Alphaproteobacteria [35, 64]. From the benthic marine environment, this lineage was represented by core members *Labrenzia*, *Roseitalea*, and *Cohaesibacter*. However, little is known about the metabolism of the latter genus, and these functions have not been experimentally reported. Among micronutrients and trace metals made bioavailable to microeukaryotes by bacteria, iron (Fe) is considered highly prominent. In bacteria, Fe can be captured by secretion of siderophores like vibrioferrin in *Marinobacter* [65] or massiliachelin in *Massilia* [66], both identified as core members of the *Prorocentrum* microbiome in this study. Transfer of sequestered bioavailable Fe to the dinoflagellate may be a critical function under Fe-limited environmental conditions. Bacterial metabolic activity in xenic dinoflagellate cultures and the known preference for ammonium ion (NH_4_^+^) instead of nitrate (NO_3_^-^) uptake for growth and toxin biosynthesis in *Prorocentrum* [67] demonstrates a potentially critical role of the bacterial community in nutrient recycling and conversion of inorganic to organic N and P as growth substrates. Although N is generally considered the primary limiting macronutrient for eukaryotic microalgae growth in marine ecosystems, in certain circumstances (e.g. in Norwegian fjords and the Ligurian Sea, Mediterranean), inorganic P may be scarce and recalcitrant and hence become the limiting nutrient (reviewed in Cembella et al. [68, 69]). In any case, bacterial mobilization of inorganic P-nutrients and conversion to low molecular weight organic P compounds which may be readily assimilated by eukaryotic microalgae are a major factor in both freshwater and marine systems [69]. In the marine environment, a possible role for *Massilia* could be the solubilization of phosphate as has been reported for terrestrial representatives [70].

The third category of known functions among bacterial core members covers a variety of secondary metabolism pathways. These functions likely serve as co-adaptation advantages and may change depending on the environment or during the growth stages or changes in the health status of the microalga in co-culture. In early stationery and senescence growth phases in culture, microalgal cells tend to become progressively leakier, releasing cell contents as bacterial load increases. Nevertheless, even during early growth phases, microalgae typically release low molecular weight molecules, such as amino acids or organic carbon [49]. High cell abundances of *Marivita* have been found to be associated with the lag phase of dinoflagellate *Margalefidinium* (*Cochlodinium*) *polykrikoides*, suggesting their participation as growth promoters through the production of vitamins [17]. *Marivita* has been found in association with epibenthic [38] and planktonic [41] dinoflagellates, and coccolithophorids [71]. High *Marivita* cell abundances were also part of the microbial community of the planktonic dinoflagellate *Lingulodinium polyedra*, a B1 and B12-vitamin auxotroph [72]. Machinery for the production of energy reservoirs was identified in *Cohaesibacter* and *Mameliella*. In these bacteria, stored poly-β-hydroxybutyrate (PHB) granules can be metabolized when common energy sources are in short supply [73, 74]. Moreover, eukaryotic microalgae can accumulate polycyclic aromatic hydrocarbons directly from seawater, and core members *Mameliella* and *Algiphilus* can metabolize a series of aromatic compounds [74, 75]. In fact, *Algiphilus* cell abundance in cultures of *Lingulodinium* increased after phenanthrene enrichment [76]. Finally, antibiotic production by bacteria is a strategy to deter the co-settling of competitors, including pathogenic bacteria, and microeukaryotes. *Mameliella* strains may produce the antibiotics bacteriocins and microcins [77], whereas the secondary pigment violacein synthesized by *Massilia* and explored as an anti-cancerogenic agent [78] is also highly toxic to bactivorous nanoflagellates.

### Role of bacterial community diversity in the regulation of toxigenicity

Studies of the bacterial community in non-toxigenic and toxigenic strains originating from the same sites may help elucidate if specific bacterial profiles promote the toxigenicity of the host dinoflagellate. *Prorocentrum* strains from diverse locations in Mexico were previously shown [4, 16] to produce mainly the linear polyketide okadaic acid (OA) and its respective diol-ester (OA-D8), but the relationship with associated bacteria remained unclear. The single *P*. *hoffmannianum* strain PA85 yielded only four DST analogs, with approximately equimolar OA and OA-D8 as dominant analogs, followed by DTX1>DTX1-D8. This composition is unusual compared to the *P*. *lima* strains in this study, but the total cell toxin composition (fmol cell^-1^) could not be distinguished from other *Prorocentrum* strains from the Veracruz reef system.

In several cases, benthic *Prorocentrum* culture conditions have been optimized for maximum DST production [79], and even studied through the cell cycle [67]. The effects of toxigenicity on the bacterial assemblages, however, are not usually investigated, with rare exceptions [16, 19]. Previous studies report that depletion of bacteria by antibiotics did not affect the toxin production in *P*. *lima* cultures [16], providing evidence that complete DST synthesis by the dinoflagellate is not bacteria-dependent [80], nor subject to associated bacterial polyketide synthase (PKS) genes [47]. Inorganic nutrient limitation in *P*. *lima* may lead to an increase in DST cell quota due to uncoupling of toxin biosynthesis from growth [67], but with negligible effect on the DST toxin composition profile. In all cases, the experiments were conducted with non-axenic *Prorocentrum* cultures, therefore the nutrient-dependent role of bacteria is unknown. The question remains open of whether effects of associated bacteria on toxigenicity of *Prorocentrum* cells are indirectly manifested through general effects on growth and metabolism, or via direct transcriptomic or post-transcriptomic regulation of dinoflagellate PKS activity.

The decision to remove the only non-toxigenic strain *P*. *rhathymum* PA20 from specific analyses was justified by the fact that not a single bacterial taxon in its microbiome stood out by absence of toxigenicity. Nevertheless, among the bacterial taxa that were represented in higher relative abundance in PA20 compared to the toxigenic ones included functions related to the metabolism of C1 compounds (e.g., methylamine and methanol) by *Methylophaga* representatives [81, 82], or the inability to reduce nitrate by members of the genus *Hoeflea* isolated from a *P*. *lima* culture [83]. In the current analysis herein, the most abundant DSTs (OA and OA-D8) produced by these *Prorocentrum* strains were not significantly associated with the presence of particular bacteria. This result agrees with the growth data and DST production by a different set of *Prorocentrum* strains from the Gulf of Mexico and the Caribbean [16]. Only two closely related DST analogs (DTX1a and DTX1a-D8) presented significant correlations with bacterial ASVs. Taking into account that maximum taxonomic assignment of ASVs is at the genus level, these significantly-correlated taxa indicate that different bacterial species may be involved in the core assemblage than in the correlation with toxin production. Among ASVs correlating with toxin production are taxa from recognized families or genera with algicidal activity (Alteromonadaceae, *Kordia*, and Saprospiraceae), suggesting allelochemical interactions between these groups. As both the toxins and the bacteria involved in this correlation are present in low abundance, a bacteriostatic effect is plausible.

## Conclusions

A more diverse bacterial community than previously characterized was detected by HTS techniques in xenic *Prorocentrum* cultures and apparently is maintained in both operationally defined compartments–free living in the medium (M) and in close association with the host (H). These H- and M-compartments were similar in composition of the bacterial microbiome. The *Prorocentrum* microbiota is partially structured based on the location of origin and may vary depending on the collection time (season). The dominant classes in strains from three disjunct locations within Mexican coastal waters were Alphaproteobacteria, Gammaproteobacteria, and Bacteroidia. The Alphaproteobacteria members of the genera *Labrenzia* and *Roseitalea* were the most abundant in the dinoflagellate-culture conditions in this study. Along with other seven genera, they comprised the *Prorocentrum* core members. Known interactions and metabolism of the core members, supported by inferred metabolism, suggest several pathways of mutualistic interaction covering carbon acquisition, nutrient exchange, and secondary metabolism of bioactive compounds. Together, the known functions of core members and the pathways to which they contribute show a variety of strategies available for a successful mutualistic relationship between the dinoflagellate host and its microbiota. Axenic dinoflagellate cultures and a variety of bacterial isolates will be required to design and perform experiments for the elucidation of specific interactions.

## Supporting information

S1 Fig Representation of the compartments analyzed from the dinoflagellate host (H) and culture medium (M) fractions. A *Prorocentrum* cell is shown surrounded by the bacterial community from these two compartments. The host-associated (H) bacteria are endosymbiotic and/or bound to the dinoflagellate cell and included within the phycosphere (orange shading) whereas bacteria within the dinoflagellate culture-medium (M) are free-living or only loosely associated with the dinoflagellate.
(PDF)

S2 Fig Bacterial community sequencing effort. Rarefaction curves of V3-V4 16S rRNA gene amplicon sequencing data processed into ASVs from bacteria of 11 *Prorocentrum* strains analyzed as the community directly associated with the dinoflagellate host (H) (top) and the free-living bacteria in the medium (M) (bottom).
(PDF)

S3 Fig Top abundant bacterial genera in *Prorocentrum* cultures. Relative abundances per sample are shown at the genus level for the top 20 abundant genera in the fraction associated with the host (H) or free-living in the culture medium (M). Taxa labels include the order name and the genus name when possible, otherwise the last assigned level is used (f = family, p = phylum). Location of dinoflagellate strain origin is indicated on the top.
(PDF)

S4 Fig Determination of *Prorocentrum* core bacterial members. A combination of high prevalence (tile color, 10 strains = 100%) and relative abundance (column-wise) were used to define the bacterial core members (indicated by black dots). ASV identifiers and associated taxonomy are shown at the left.
(PDF)

S5 Fig Radar plot of the compositional toxigenic profile of *Prorocentrum* strains. Distribution of the relative DST composition (% molar) of 10 *Prorocentrum* strains with different location of origin. OA, okadaic acid; OA-D8, okadaic acid diol-ester; DTX1, dinophysistoxin 1; DTX1-D8, dinophysistoxin 1 diol-ester; DTX1a, undescribed DTX1 isomer; DTX1a-D8, undescribed DTX1a isomer.
(PDF)

S1 Table Summary data on *Prorocentrum* species and strains, location of origin, transfer cycles, toxin composition, and associated bacterial sequencing and data analysis by fraction (NA, not available). (XLSX)

S2 Table Contribution of core ASVs to major KEGG pathways. Result of a Mann-Whitney test (with Bonferroni correction, ns = non-significant) between contribution of core and non-core ASVs.
(XLSX)
