# Supplementary material for: Functional diversity of bacterial microbiota associated with the toxigenic benthic dinoflagellate Prorocentrum
Source: PLoS One. 2024 Jul 16;19(7):e0306108. doi: 10.1371/journal.pone.0306108 (PMC11251618; doi:10.1371/journal.pone.0306108)
Supplement: S1 Fig — A Prorocentrum cell is shown surrounded by the bacterial community from these two compartments. The host-associated (H) bacteria are endosymbiotic and/or bound to the dinoflagellate cell and included within the phycosphere (orange shading) whereas bacteria within the dinoflagellate culture-medium (M) are free-living or only loosely associated with the dinoflagellate. (PDF) [file pone.0306108.s001.pdf]

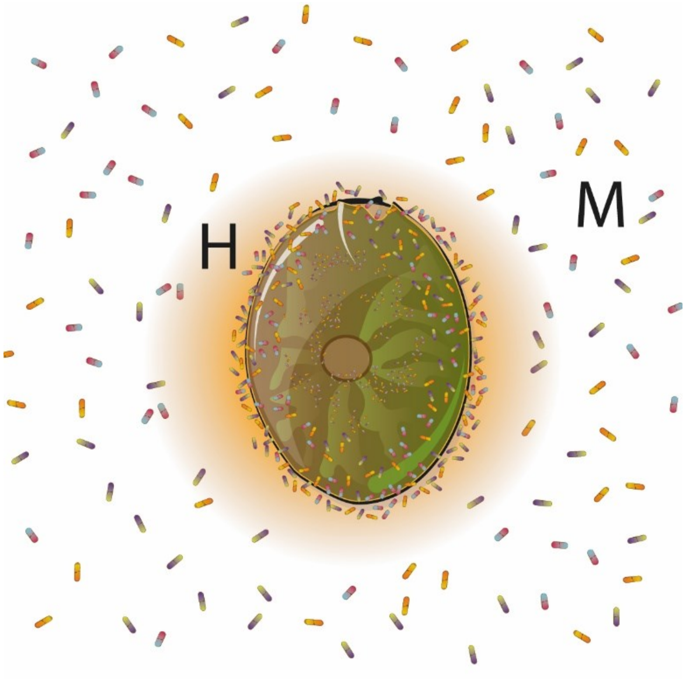

Supplementary Figure 1. Representation of the compartments analyzed from the dinoflagellate host (H) and culture medium (M) fractions. A *Prorocentrum* cell is shown surrounded by the bacterial community from these two compartments. The host-associated (H) bacteria are endosymbiotic and/or bound to the cell and included within the phycosphere (orange shading) whereas bacteria within the dinoflagellate culture-medium (M) are free-living or only loosely associated with the dinoflagellate.
