# Supplementary material for: Functional diversity of bacterial microbiota associated with the toxigenic benthic dinoflagellate Prorocentrum
Source: PLoS One. 2024 Jul 16;19(7):e0306108. doi: 10.1371/journal.pone.0306108 (PMC11251618; doi:10.1371/journal.pone.0306108)
Supplement: S2 Fig — Rarefaction curves of V3-V4 16S rRNA gene amplicon sequencing data processed into ASVs from bacteria of 11 Prorocentrum strains analyzed as the community directly associated with the dinoflagellate host (H) (top) and the free-living bacteria in the medium (M) (bottom). (PDF) [file pone.0306108.s002.pdf]

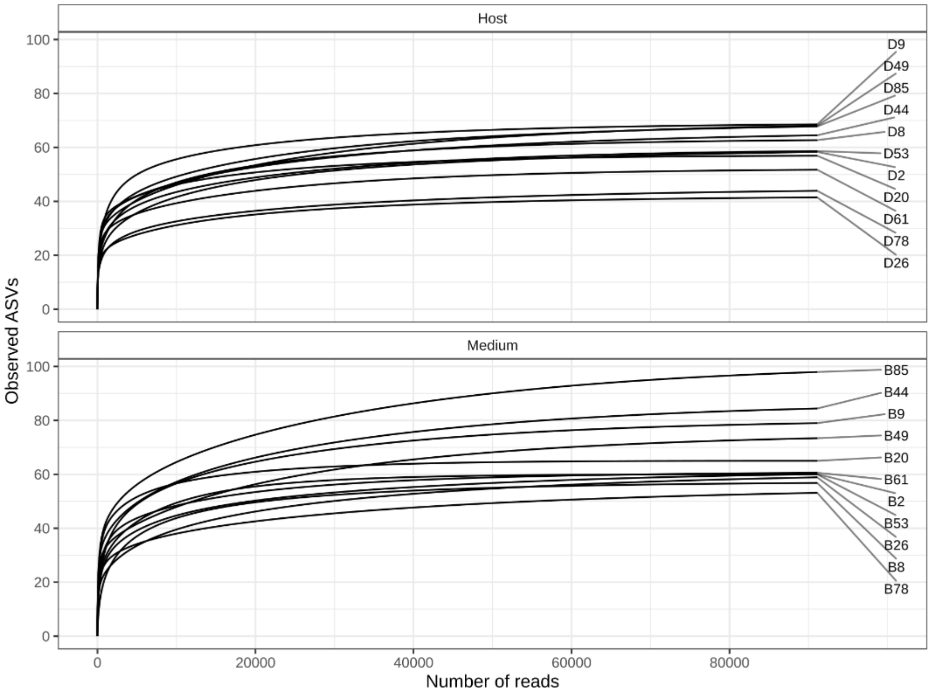

S2 Fig. Bacterial community sequencing effort. Rarefaction curves of V3-V4 16S rRNA gene amplicon sequencing data processed into ASVs from bacteria of 11 *Prorocentrum* strains analyzed as the community directly associated with the dinoflagellate host (H) (top) and the free-living bacteria in the medium (M) (bottom).
