# Supplementary figures and images for: Functional diversity of bacterial microbiota associated with the toxigenic benthic dinoflagellate Prorocentrum
Source: PLoS One. 2024 Jul 16;19(7):e0306108. doi: 10.1371/journal.pone.0306108 (PMC11251618; doi:10.1371/journal.pone.0306108)

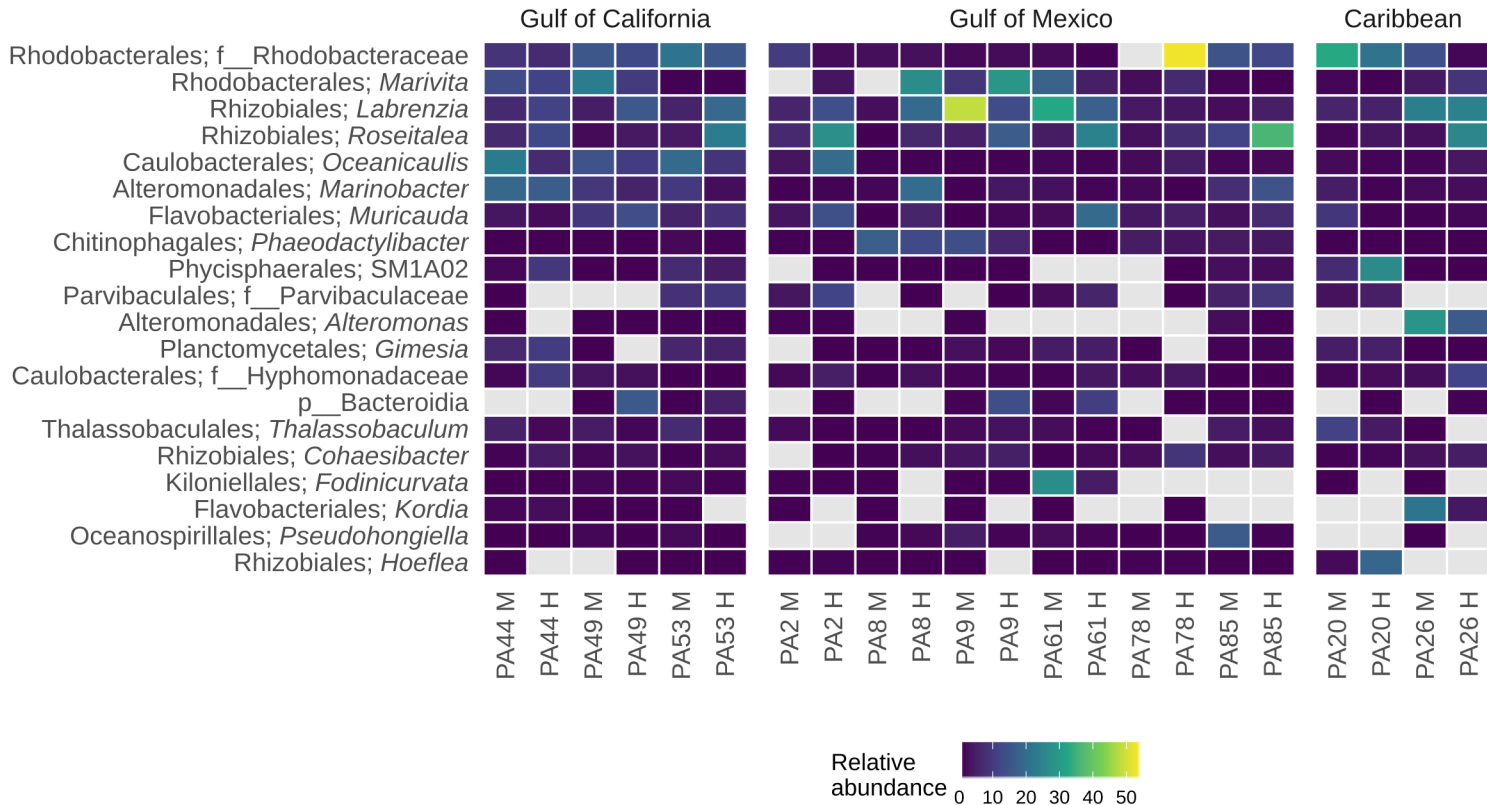

Supplement: S3 Fig — Relative abundances per sample are shown at the genus level for the top 20 abundant genera in the fraction associated with the host (H) or free-living in the culture medium (M). Taxa labels include the order name and the genus name when possible, otherwise the last assigned level is used (f = family, p = phylum). Location of dinoflagellate strain origin is indicated on the top. (PDF) [file pone.0306108.s003.pdf]
