# Supplementary material for: Functional diversity of bacterial microbiota associated with the toxigenic benthic dinoflagellate Prorocentrum
Source: PLoS One. 2024 Jul 16;19(7):e0306108. doi: 10.1371/journal.pone.0306108 (PMC11251618; doi:10.1371/journal.pone.0306108)
Supplement: S4 Fig — A combination of high prevalence (tile color, 10 strains = 100%) and relative abundance (column-wise) were used to define the bacterial core members (indicated by black dots). ASV identifiers and associated taxonomy are shown at the left. (PDF) [file pone.0306108.s004.pdf]

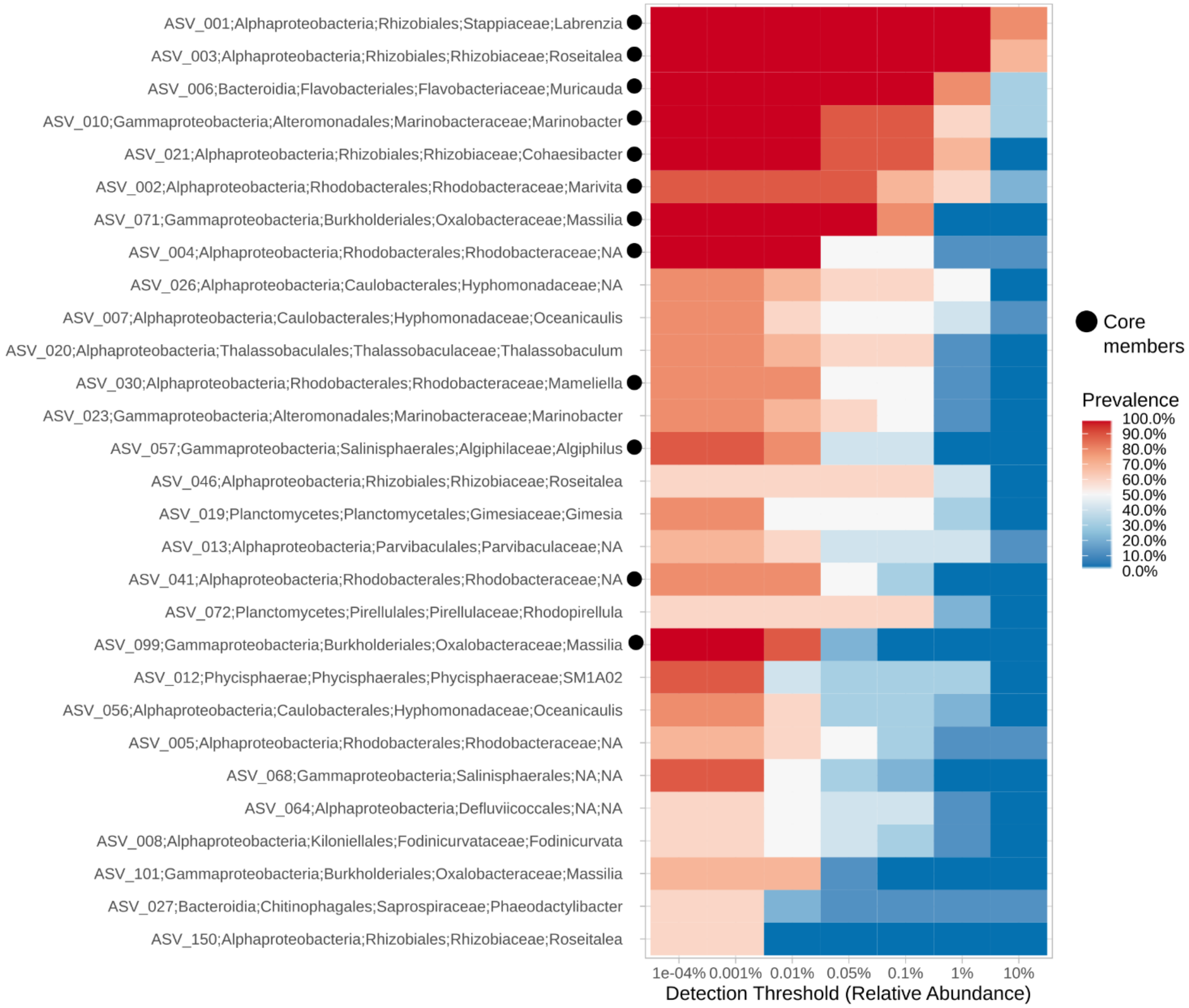

S4 Fig. Determination of *Prorocentrum* core bacterial members. A combination of high prevalence (tile color, 10 strains = 100%) and relative abundance (column-wise) were used to define the bacterial core members (indicated by black dots). ASV identifiers and associated taxonomy are shown at the left.
