# Supplementary material for: Functional diversity of bacterial microbiota associated with the toxigenic benthic dinoflagellate Prorocentrum
Source: PLoS One. 2024 Jul 16;19(7):e0306108. doi: 10.1371/journal.pone.0306108 (PMC11251618; doi:10.1371/journal.pone.0306108)
Supplement: S5 Fig — Distribution of the relative DST composition (% molar) of 10 Prorocentrum strains with different location of origin. OA, okadaic acid; OA-D8, okadaic acid diol-ester; DTX1, dinophysistoxin 1; DTX1-D8, dinophysistoxin 1 diol-ester; DTX1a, undescribed DTX1 isomer; DTX1a-D8, undescribed DTX1a isomer. (PDF) [file pone.0306108.s005.pdf]

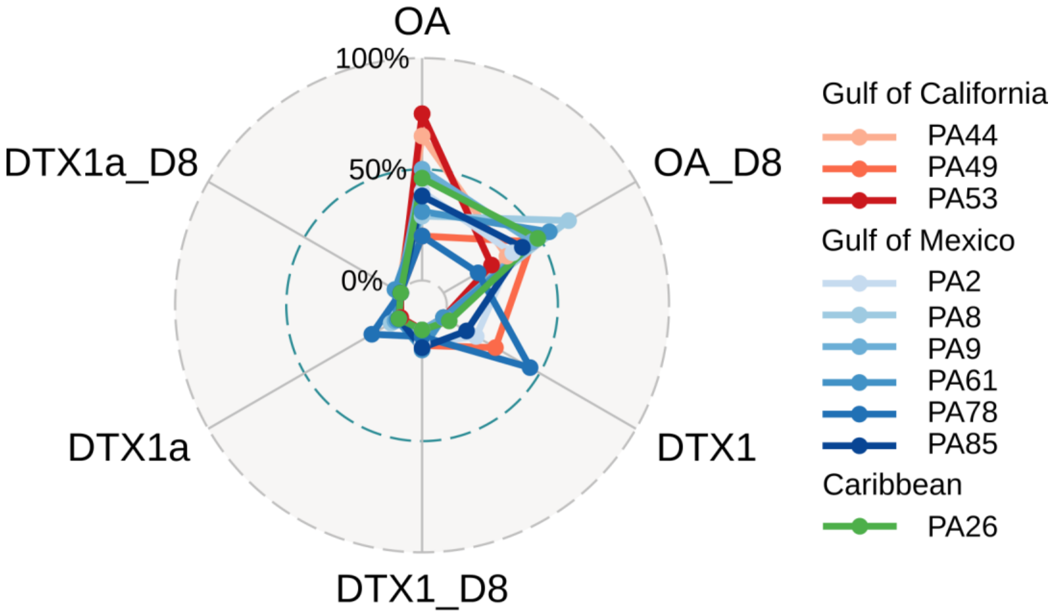

S5 Fig. Radar plot of the compositional toxigenic profile of *Prorocentrum* strains. Distribution of the relative DST composition (% molar) of 10 *Prorocentrum* strains with different location of origin. OA, okadaic acid; OA-D8, okadaic acid diol-ester; DTX1, dinophysistoxin 1; DTX1-D8, dinophysistoxin 1 diol-ester; DTX1a, undescribed DTX1 isomer; DTX1a-D8, undescribed DTX1a isomer.
